# Pressure-induced structural changes cause large enhancement of photoluminescence in halide perovskites: a quantitative relationship

**DOI:** 10.1093/nsr/nwab041

**Published:** 2021-03-09

**Authors:** Alexandra Navrotsky

**Affiliations:** Center for Materials of the Universe, Arizona State University, USA

Halide perovskites containing both organic and inorganic cations are receiving extensive attention for their promising optoelectronic properties, including photoluminescence (PL) [1,2]. Tuning these properties for specific applications and designing and synthesizing perovskites with optimized properties are major goals of materials science. These goals will be achieved faster and more economically by harnessing a fundamental understanding of structure–property relations, rather than by random searches for better materials. Chemical composition, temperature and pressure are the major ‘knobs’ one can turn to adjust properties such as conductivity and bandgap [1,3]. Each of these variables has the potential to change interatomic distances, electronic and magnetic structure, and local and long-range symmetry—all determinants of physical properties. However, pressure, a variable that comes more easily to mind in the geologic than in the materials community, has not, up to now, been utilized frequently enough to systematically tune halide perovskites.

**Figure 1.**
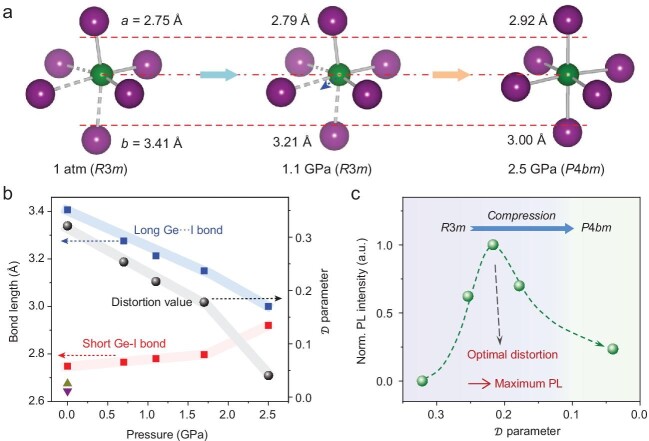
(a) Distortion and off-centering in CsGeI_3_ perovskite as a function of pressure. (b) Bond lengths and distortion parameter as a function of pressure. (c) PL intensity as a function of distortion parameter, clearly showing an optimal value [4].

The pioneering work by Lü *et al*. [4], involving collaboration among geoscientists and materials scientists, reveals a quantitative structure–property relationship in the halide perovskites. By applying pressures up to 4 GPa (40 000 atmospheres) to highly distorted methylammonium germanium iodide (CH_3_NH_3_GeI_3_), which permits a high structural tunability, one can both greatly increase the intensity and change the wavelength of PL. These effects are shown to be related to the off-centering distortions in the octahedral sites of the perovskite as determined by parallel *in situ* PL and crystallographic studies (Fig. 1a). Aided by first principles calculations, mechanisms are proposed for these changes in bandgap and PL intensity. Competition among several bonding factors [4,5] suggests that there may be an optimal degree of distortion for maximum PL (Fig. 1b and c).

Lü *et al.* validated their ideas on further studies of the (CH_3_NH_3_)_(1-x)_Cs_x_GeI_3_ system and of formamidinium germanium iodide, both systems synthesized at ambient pressure where the degree of off-centering is controlled by the average size of the A-site cation. They defined a distortion parameter that can be optimized to produce desirable PL properties (Fig. 1c). These new systematic correlations of PL properties with a crystallographic distortion parameter lead the way to synthesis of perovskites with controllable PL wavelength and much-enhanced PL intensity. Thus, by using high pressure as a strong ‘knob’ to change distortions and bond lengths and to gain fundamental mechanistic understanding, one can predict optimized PL perovskite compositions to be synthesized at ambient pressure for technological uses.

A similar strategy, namely utilizing high pressure to extend the variation of bond lengths and distortions and quantify their effects on desired physical properties, should apply to many other systems (not just perovskites) of potential technological interest. For example, Cheng *et al.* [6] applied ideas of pressure-induced distortions to optoelectronic oxide systems. However, a word of caution is in order. As the structures become more distorted in both halide and oxide perovskites, they tend to have lower thermodynamic stability because of mismatch in ionic sizes and differences in bonding, with chlorides more stable than bromides and bromides more stable than iodides [7]. Therefore, one is likely to encounter trade-offs between optimal optical behavior and the robustness of materials in applications, even when such materials, identified through optimizing distortion parameters, are synthesized at atmospheric pressure.

***Conflict of interest statement*.** None declared.
